# Predicting concurrent endometrial carcinoma in women with endometrial intraepithelial neoplasia without preoperative suspicion of malignancy

**DOI:** 10.1016/j.gore.2026.102236

**Published:** 2026-10-19

**Authors:** Gil Zeevi, Effi Yeoshoua, Chani Dahan, Or Bercovich, Oded Raban, Ariella Jakobson-Setton, Gad Sabah, Ran Matot, Adi Borovich, Ram Eitan

**Affiliations:** a Helen Schneider Hospital for Women, Rabin Medical Center, Petach Tikva, Israel; b Gray Faculty of Medical and Health Sciences, Tel Aviv University, Tel Aviv, Israel

**Keywords:** Endometrial intraepithelial neoplasia, Endometrial carcinoma, Nomogram, Preoperative risk assessment

## Abstract

**Objective:**

Endometrial intraepithelial neoplasia (EIN) is a premalignant lesion with variable risk of concurrent endometrial carcinoma. Identifying predictors of endometrial carcinoma in women with EIN without pathological suspicion of carcinoma may improve surgical planning.

**Methods:**

A retrospective cohort study of patients with EIN who underwent hysterectomy between 2014 and 2024. Preoperative clinical and diagnostic variables were evaluated as predictors of concurrent endometrial carcinoma and incorporated into a multivariable logistic regression model to construct a nomogram. Model discrimination was assessed using the area under the receiver operating characteristic curve. Ridge and Firth penalized regression were performed as sensitivity analyses.

**Results:**

The final cohort included 124 women, of whom 18 (14.5%) had concurrent endometrial carcinoma. Type 2 diabetes mellitus was associated with increased odds of concurrent carcinoma (adjusted OR 4.18, 95% CI 1.11–15.69). Hysteroscopy-guided sampling or dilation and curettage was associated with lower odds compared with pipelle biopsy (adjusted OR 0.11, 95% CI 0.02–0.50). A nomogram incorporating age, body mass index, type 2 diabetes mellitus, and diagnostic modality demonstrated an AUC of 0.828 (95% CI 0.725–0.931) and an optimism-corrected AUC of 0.784. Penalized sensitivity analyses yielded consistent findings.

**Conclusion:**

A simple preoperative nomogram based on routinely available clinical variables showed promising discrimination for concurrent endometrial carcinoma in women with EIN. Prospective and external validation in independent cohorts is required before clinical implementation.

## Highlights

• Concurrent carcinoma was found in 14.5% of women with EIN without preoperative pathologic suspicion of malignancy.
• Type 2 diabetes mellitus and sampling method were associated with concurrent endometrial carcinoma.
• A four-variable model showed good discrimination after internal validation.
• The model may support individualized preoperative risk assessment in women with EIN.

## 1 Introduction

Endometrial cancer is one of the most common gynecologic malignancies worldwide, with a steadily rising incidence over recent decades, largely driven by the increasing prevalence of obesity and metabolic disease (Crosbie et al., 2022; Zhong et al., 2025). Endometrial intraepithelial neoplasia (EIN) represents the premalignant counterpart of endometrioid endometrial carcinoma within a shared pathobiologic spectrum. (Mutter, 2000; Emons et al., 2015; Sobczuk and Sobczuk, 2017) However, its clinical significance extends beyond its role as a precursor lesion. A substantial proportion of patients diagnosed with EIN are found to harbor concurrent carcinoma at the time of hysterectomy, with reported rates approaching 40% (Trimble et al., 2006; Doherty et al., 2020). This frequent coexistence of premalignant and malignant disease highlights EIN as not only a marker of future cancer risk, but also a clinically relevant indicator of occult malignancy at presentation.

From a pathologic perspective, EIN represents a clonal proliferation of endometrial glands characterized by glandular crowding and cytologic features distinct from the surrounding background endometrium, consistent with early neoplastic transformation (Allison et al., 2008; Sanderson et al., 2017). The diagnosis is based on morphologic criteria including an increased gland-to-stroma ratio, cytologic demarcation, and exclusion of benign mimics and overt carcinoma (Owings and Quick, 2014). However, distinguishing EIN from well-differentiated carcinoma on preoperative endometrial sampling remains challenging (Trimble et al., 2012; Vetter et al., 2020).

EIN and endometrial carcinoma share common hormonal and metabolic risk factors, including obesity, chronic anovulation, and nulliparity (1,12). Accordingly, several preoperative factors have been evaluated as predictors of concurrent carcinoma (Levin et al., 2024; Li et al., 2025; Liang et al., 2025; Matanes et al., 2023). However, existing studies have reported inconsistent findings, and the role of prediction models in routine clinical decision-making remains uncertain. Moreover, past studies have generally evaluated heterogeneous EIN populations, including women whose preoperative pathology already raised concern for carcinoma. The residual risk of occult malignancy among women with an unequivocal diagnosis of EIN without pathologic suspicion of carcinoma remains less well characterized. Improved preoperative risk stratification in this clinically distinct population could support individualized counseling, gynecologic oncology referral, and surgical planning. Therefore, the aim of the present study was to identify preoperative clinical and diagnostic predictors of concurrent endometrial carcinoma in women with EIN without pathologic suspicion of carcinoma and to develop an internally validated prediction model to support individualized preoperative risk assessment.

## 2 Methods

### 2.1 Study design

A retrospective cohort study was conducted at a single tertiary, university-affiliated medical center between January 2014 and December 2024. Clinical data were retrieved from comprehensive electronic medical records. The study included all women with a preoperative histopathologic diagnosis of endometrial intraepithelial neoplasia (EIN) who subsequently underwent hysterectomy during the study period.

Patients managed conservatively with progestin-based therapy were excluded, as were patients whose original pathology report described EIN with features suspicious for carcinoma or included a concurrent diagnosis of carcinoma. The study cohort was restricted to cases classified as EIN on the original pathology report.

To evaluate predictors of concurrent endometrial carcinoma, patients were stratified according to final histopathology following hysterectomy into those with and without endometrial carcinoma. During the study period women with a pre-operative diagnosis of EIN did not undergo staging procedures in accordance with departmental guidelines.

### 2.2 Data collection

Preoperative variables included demographic, clinical, imaging, and diagnostic factors. Demographic and clinical variables included age at diagnosis, body mass index (BMI), gravidity, parity, menopausal status, age at menopause, and time from menopause to diagnosis. Menopausal status was determined from the medical record based on physician documentation and was defined as at least 12 consecutive months without menstruation. Age at menopause and the interval from menopause to EIN diagnosis were recorded only for postmenopausal women when the age at menopause was documented. Comorbidities included hypertension, type 2 diabetes mellitus, dyslipidemia, history of breast cancer, and smoking status. Medication exposure included hormone replacement therapy and tamoxifen use.

Preoperative assessment included transvaginal ultrasound measurement of endometrial thickness and the method of endometrial sampling. For patients who underwent more than one sampling procedure, diagnostic modality was classified according to the procedure that established the final preoperative histopathologic diagnosis used for surgical management. The interval from diagnosis to surgery was recorded. Surgical variables included operative approach and type of procedure performed.

All preoperative endometrial sampling and final hysterectomy pathology reports were independently reviewed by two investigators to confirm accurate classification of the preoperative and final histologic diagnoses. Pathologic variables included final histologic diagnosis. For patients diagnosed with endometrial carcinoma, tumor grade, FIGO stage (Berek et al., 2023), depth of myometrial invasion, cervical involvement, lymphovascular space invasion, lymph node status, and molecular markers including mismatch repair proteins, p53 status, and hormone receptor expression were collected. Additional data regarding adjuvant treatment was also recorded.

### 2.3 Development of the prediction model

Preoperative characteristics were compared between patients with and without concurrent endometrial carcinoma on final hysterectomy pathology. Univariable analyses were performed to assess the association between preoperative variables and concurrent carcinoma. Variables entered into the multivariable model included prespecified predictors based on clinical relevance and previous studies (age, body mass index, and type 2 diabetes mellitus), together with diagnostic modality, which was associated with concurrent carcinoma in the univariable analysis. Given the small number of patients who underwent dilation and curettage, hysteroscopy-guided sampling and dilation and curettage were combined into a single category for multivariable modeling and compared with pipelle biopsy. Age was evaluated using two specifications, as a continuous variable and dichotomized at ≥65 years. A multivariable logistic regression model incorporating these four variables was developed, and a nomogram was derived from the final model to estimate the probability of concurrent endometrial carcinoma. To assess the robustness of the model estimates in this relatively small cohort, sensitivity analyses using ridge-penalized regression and Firth logistic regression were performed. As an additional sensitivity analysis, the model was repeated after including patients whose preoperative pathology raised suspicion of carcinoma and who were excluded from the primary cohort.

### 2.4 Statistical analysis

Categorical variables were presented as frequencies and percentages. The normality of continuous variables was checked using the Shapiro-Wilk test, histograms, and Q-Q plots. Continuous variables with a normal distribution are expressed as means with standard deviations (± SD). Variables that were skewed are presented as medians with interquartile ranges (IQRs). Continuous variables were compared using the Welch's *t*-test or the Mann-Whitney *U* test as appropriate. Categorical variables were analyzed using the Chi-square test or Fisher's exact test as appropriate. Multivariable logistic regression was performed using complete case analysis, and adjusted odds ratios (ORs) with 95% confidence intervals (CIs) were reported. Model discrimination was assessed using the area under the receiver operating characteristic curve (AUC), with 95% CIs calculated using the DeLong method. Model calibration was assessed graphically using bootstrap-corrected calibration curves and quantitatively using the calibration slope. Internal validation was performed using 1000 bootstrap resamples, with the full model-fitting procedure repeated in each resample, and an optimism-corrected AUC was derived from Somers' Dxy. Ridge-penalized regression and Firth logistic regression were performed as sensitivity analyses. All tests were two-sided, with *p* < 0.05 considered statistically significant. Analyses were performed using R version 4.5.1 (R Core Team, 2025).

### 2.5 Ethics

The study was approved by the local institutional review board (approval no.0037–25). Informed consent was waived due to the retrospective study design.

## 3 Results

During the study period, 149 women were diagnosed with endometrial intraepithelial neoplasia (EIN) at our institution. Ten women underwent conservative management with progestin therapy and were therefore excluded from the analysis. In addition, fifteen patients were excluded because preoperative biopsy showed EIN with suspicion for endometrial carcinoma. Final hysterectomy pathology confirmed endometrial carcinoma in 12 of these 15 women (80%). The final study cohort, included 124 women with a preoperative diagnosis of EIN without suspicion of carcinoma who subsequently underwent hysterectomy. Concurrent endometrial carcinoma was identified in 18 patients (14.5%), while 106 (85.5%) had no evidence of carcinoma on final hysterectomy pathology. (Supplementary Fig. S1).

Baseline clinical characteristics were largely comparable between groups (Table 1). Median age at EIN diagnosis was similar in patients with and without concurrent carcinoma (66 vs 62 years; *p* = 0.779). The proportion of postmenopausal women did not differ between groups (72% vs 77%; *p* = 0.763). Median body mass index tended to be higher among women with carcinoma (34.5 vs 30.5 kg/m^2^; *p* = 0.071), although the difference did not reach statistical significance. Other clinical factors including parity, hypertension, dyslipidemia, prior breast cancer, Tamoxifen use, and smoking status were also similar between groups. Type 2 diabetes mellitus was more frequent among women with carcinoma (33% vs 13%; *p* = 0.077), although this difference did not reach statistical significance. The distribution of diagnostic methods differed significantly between groups (*p* = 0.013), with pipelle biopsy performed in 33.3% of women with concurrent carcinoma versus 8.5% of those without carcinoma, hysteroscopic sampling in 61.1% versus 86.8%, and dilation and curettage in 5.6% versus 4.7%, respectively. Endometrial thickness was available for 85 of 124 patients (68.5%), including 73 of 106 women without concurrent carcinoma and 12 of 18 women with carcinoma. Median endometrial thickness did not differ significantly between groups (11.0 vs 8.0 mm, *p* = 0.125). (Table 1).

**Table 1:** Characteristics of patients according to final pathology.

Variable	No concurrent carcinoma *N* = 106	Endometrial carcinoma *N* = 18	*p*-value
Age at diagnosis, years	62.0 (51.0–68.0)	66.0 (54.0–66.0)	0.779
Age at diagnosis ≥ 65	45 (42%)	12 (67%)	0.099
Body mass index (kg/m2)	30.47 (26.52–34.68)	34.53 (28.79–36.92)	0.071
Parity	2.00 (1.00, 3.00)	2.50 (1.00, 4.00)	0.681
Gravidity	3.00 (2.00, 4.00)	3.00 (1.00, 4.00)	0.754
Menopause at diagnosis	82 (77%)	13 (72%)	0.763
Age at menopause	50.50 (47.00–55.00)	50.00 (48.00–50.00)	0.303
Time from menopause to diagnosis, months	13.00 (6.00–21.00)	16.00 (15.00–17.50)	0.352
Hypertension	40 (38%)	8 (44%)	0.589
Dyslipidemia	43 (42%)	8 (44%)	0.831
Type 2 diabetes mellitus	14 (13%)	6 (33%)	0.077
History of breast cancer	13 (12%)	0 (0%)	0.212
Tamoxifen use	10 (9.4%)	0 (0%)	0.355
Smoking	3 (3.2%)	1 (6.3%)	0.468
Endometrial thickness, mm*	11.0 (7.5–14.0)	8.0 (4.55–11.0)	0.125
Diagnostic method			0.013
Pipelle	9 (8.5%)	6 (33%)	
Hysteroscopy	92 (87%)	11 (61%)	
Dilation and curettage	5 (4.7%)	1 (5.6%)	
Interval diagnosis to surgery, days	98.0 (66.0–135.0)	90.0 (71.0–127.0)	0.957
Minimally invasive surgery	91 (85.8)	14 (77.8)	0.476
Open surgery	15 (14.2)	4 (22.2)	0.476

*Endometrial thickness was measured by transvaginal ultrasound and was available for 85 of 124 patients (68.5%). Data are presented as median (IQR) or n (%). Percentages are calculated based on available data for each variable; denominators may therefore vary because of missing data

Surgical characteristics were similar between groups (Table 1). The majority of procedures were performed using minimally invasive techniques, and the surgical approach did not differ significantly between patients with and without carcinoma (78% vs 86%; *p* = 0.476). The median interval between EIN diagnosis and hysterectomy was also comparable between groups (90 vs 98 days; *p* = 0.957).

Among the 18 women with concurrent Endometrial carcinoma, all tumors were Endometrioid adenocarcinomas. Seventeen tumors (94.4%) were grade 1 and one (5.6%) was grade 2. Nearly all tumors were early stage, with one patient (5.6%) classified as FIGO 2023 stage IA1, 16 (88.9%) as stage IA2, and one (5.6%) as stage IB. No patient had cervical involvement, lymphovascular space invasion, or abnormal p53 expression. One tumor (5.6%) demonstrated mismatch repair deficiency due to loss of MLH1/PMS2 expression, and all tumors were estrogen receptor and progesterone receptor positive (Table 2).

**Table 2:** Pathologic characteristics of patients with concurrent Endometrial carcinoma (N = 18).

Variable	Value
	n (%)
Histologic subtype	
Endometrioid adenocarcinoma	18 (100)
Tumor grade	
Grade 1	17 (94.4)
Grade 2	1 (5.6)
Grade 3	0 (0)
FIGO 2023 stage	
IA1	1 (5.6)
IA2	16 (88.9)
IB	1 (5.6)
Pathologic features	
Myometrial invasion <50%	17 (94.4)
Myometrial invasion ≥50%	1 (5.6)
Isthmic/cervical involvement	0 (0)
Lymphovascular space invasion	0 (0)
Immunohistochemical profile	
Abnormal p53 expression	0 (0)
Mismatch repair deficiency	1 (5.6)
Estrogen receptor positive	18 (100)
Progesterone receptor positive	18 (100)

Two multivariable models were evaluated, each including age, BMI, type 2 diabetes mellitus, and diagnostic modality. Given the substantial proportion of missing endometrial thickness measurements and the resulting reduction in the number of outcome events available for complete-case multivariable analysis, endometrial thickness was not included in the primary prediction model.The models differed only in the specification of age: Model 1 included age as a continuous variable, whereas Model 2 included age dichotomized at ≥65 years. Model 2 demonstrated numerically better discrimination, with an apparent AUC of 0.828 (95% CI 0.725–0.931), compared with 0.795 (95% CI 0.678–0.913) for Model 1. After 1000 bootstrap resamples, the optimism-corrected AUC were 0.784 and 0.748, respectively. The difference in apparent AUC between the models was not statistically significant (ΔAUC = 0.033; DeLong *p* = 0.175). Given the similar discrimination of the two age specifications and the greater clinical interpretability of a categorical age threshold, Model 2 was selected for nomogram presentation.

In the final model, type 2 diabetes mellitus was associated with increased odds of concurrent endometrial carcinoma (adjusted OR 4.18, 95% CI 1.11–15.69; *p* = 0.034). Hysteroscopy-guided sampling or dilation and curettage was associated with lower odds of concurrent carcinoma compared with pipelle biopsy (adjusted OR 0.11, 95% CI 0.02–0.50; *p* = 0.004). Age ≥ 65 years was not significantly associated with concurrent carcinoma (adjusted OR 3.97, 95% CI 0.98–16.10; *p* = 0.054), whereas BMI was not significantly associated with the outcome (adjusted OR 1.07, 95% CI 0.98–1.17; *p* = 0.112) (Table 3B).

**Table 3:** Multivariable logistic regression for concurrent Endometrial carcinoma.

A. Model 1			
Variable	Adjusted OR	95% CI	*p* value
			
Age (continuous)	1.02	0.95–1.10	0.521
Diagnostic modality⁎	0.14	0.04–0.58	0.006
Body mass index (kg/m^2^)	1.07	0.99–1.16	0.097
Type 2 diabetes mellitus	4.39	1.15–16.76	0.031
B. Model 2			
Age ≥ 65 years	3.97	0.98–16.10	0.054
Diagnostic modality⁎	0.11	0.02–0.50	0.004
Body mass index (kg/m2)	1.07	0.98–1.17	0.112
Type 2 diabetes mellitus	4.18	1.11–15.69	0.034

⁎ Diagnostic modality: Hysteroscopy or Dilation and curettage vs Pipelle Model 2 demonstrated numerically higher discrimination than Model 1 (AUC 0.828 vs 0.795; ΔAUC 0.033; DeLong *p* = 0.175), with optimism-corrected AUC of 0.784 and 0.748, respectively

A nomogram incorporating these four variables was developed to estimate the individual probability of concurrent endometrial carcinoma (Fig. 1). The apparent AUC of the final model was 0.828 (95% CI 0.725–0.931) (Fig. 2), and the optimism-corrected AUC following 1000 bootstrap resamples was 0.784. Sensitivity analyses using ridge-penalized and Firth logistic regression were performed to assess the robustness of the model estimates (Supplementary Table S1). In the Firth analysis, type 2 diabetes mellitus remained associated with increased odds of concurrent carcinoma (OR 3.88, 95% CI 1.09–13.85), whereas hysteroscopy-guided sampling or dilation and curettage remained associated with lower odds compared with pipelle biopsy (OR 0.13, 95% CI 0.03–0.53). Ridge penalization improved model calibration, with the bootstrap-corrected calibration slope increasing from 0.76 in the unpenalized model (Supplementary Fig. S2) to 1.04 in the ridge-penalized model.

**Fig. 1:**
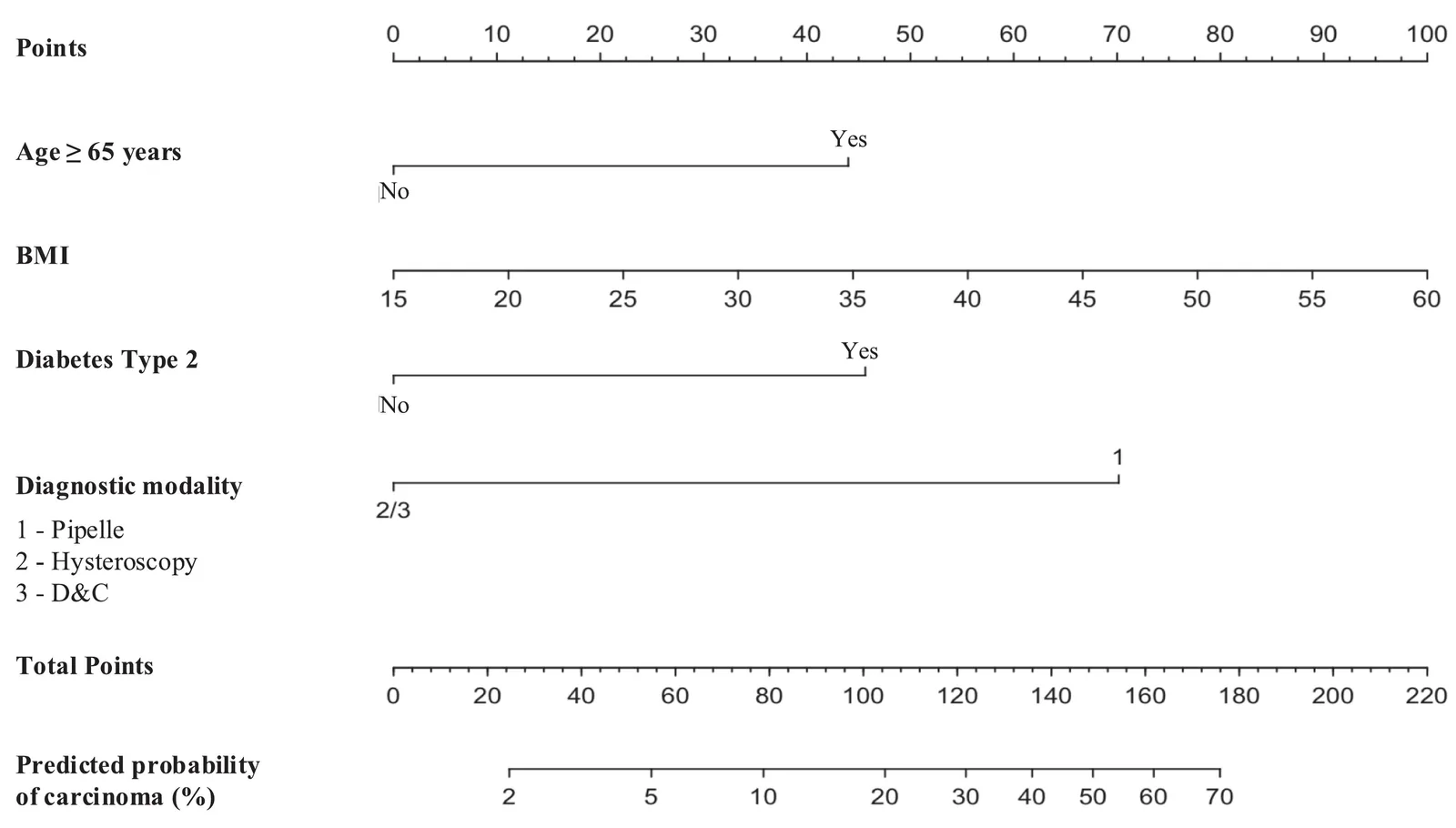
A nomogram for predicting Endometrial Carcinoma in patients with Endometrial Intraepithelial Neoplasia.

**Fig. 2:**
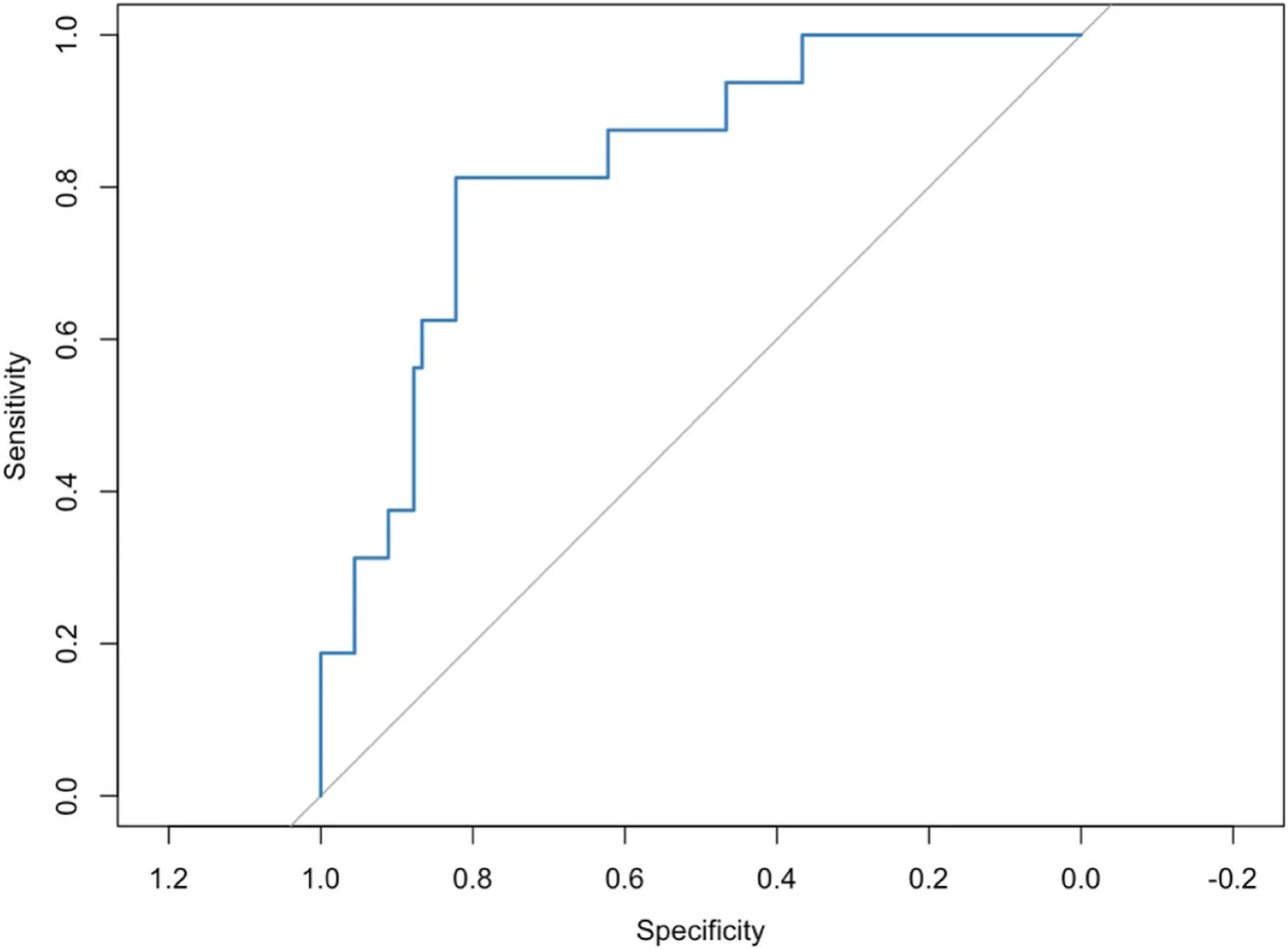
Receiver operating characteristic (ROC) curve of the nomogram for predicting concurrent endometrial carcinoma.

In an additional sensitivity analysis, the model was repeated after including the 15 women with preoperative pathology suspicious for carcinoma who had been excluded from the primary cohort. The model retained discriminatory ability, with an optimism-corrected AUC of 0.706 compared with 0.784 in the primary cohort. Effect estimates for type 2 diabetes mellitus and diagnostic modality remained consistent across both cohorts. The conventional logistic regression model was retained for nomogram development, while the penalized models were used to assess the robustness and stability of the model estimates.

## 4 Discussion

In this retrospective cohort study, we developed a simple preoperative nomogram based on routinely available clinical variables to estimate the probability of concurrent endometrial carcinoma in women with endometrial intraepithelial neoplasia without preoperative pathological suspicion of carcinoma. The model demonstrated promising discrimination following internal validation. Given the relatively small cohort and limited number of outcome events, these findings should be interpreted cautiously and considered preliminary.

Individualized preoperative risk assessment is increasingly recognized as an important component in the management of women with EIN, as the presence of concurrent endometrial carcinoma may influence patient counseling, referral to a gynecologic oncologist, surgical planning, and consideration of nodal assessment (*Obstet. Gynecol.*, 2023; Concin et al., 2025). However, accurately estimating this risk remains challenging because the reported prevalence of concurrent carcinoma varies considerably across studies (Doherty et al., 2020; *Obstet. Gynecol.*, 2023). Evidence also highlights the importance of detailed preoperative histopathologic characterization. A multicenter external validation study demonstrated substantial differences in the risk of concurrent carcinoma according to preoperative histopathologic characteristics, supporting the concept that women with EIN represent a heterogeneous population with clinically meaningful differences in underlying malignancy risk (Restaino et al., 2026).

Previous studies have explored preoperative prediction of concurrent endometrial carcinoma in women with EIN using a variety of approaches. Vetter et al. identified age and endometrial thickness as important preoperative predictors, whereas Matsuo et al. identified older age, obesity, diabetes mellitus, and complex atypical hyperplasia as independent predictors of concurrent carcinoma and demonstrated a stepwise increase in malignancy risk with accumulation of these factors (Vetter et al., 2020; Matsuo et al., 2015). More recent models have incorporated imaging and biomarker variables. A nomogram combining endometrial thickness ≥ 15 mm, menopausal status, hypertension, serum HE4, and endometrial blood flow achieved an AUC of 0.68 in its training cohort and 0.78 in an internal validation cohort (Liang et al., 2025), and another study identified BMI ≥35 kg/m^2^, postmenopausal status, and endometrial thickness ≥ 14 mm as independent predictors (Çaltek et al., 2025). These findings further support the multifactorial nature of concurrent carcinoma risk. Although endometrial thickness has emerged as an important predictor across several studies, measurements were unavailable in nearly one-third of our cohort, limiting its use in the multivariable model. Our study complements these approaches by presenting a simple nomogram based on routinely available preoperative clinical and diagnostic variables, without requiring additional biomarkers or imaging-based parameters.

Beyond risk estimation itself, preoperative stratification may influence surgical planning. Peters et al. prospectively evaluated a risk-based algorithm using endometrial thickness and pathology findings to guide sentinel lymph node assessment in women with EIN (Peters et al., 2026). In contrast, our study excluded women whose preoperative pathology already raised suspicion for carcinoma and focused specifically on an unequivocal EIN population. Our model therefore addresses the residual risk of concurrent carcinoma when the pathology itself does not suggest malignancy.

The variables incorporated into the nomogram represent complementary aspects of preoperative risk assessment. Patient-related characteristics, including age, body mass index, and diabetes mellitus status, were combined with the diagnostic modality used for endometrial sampling. Obesity and type 2 diabetes mellitus are well-established risk factors for endometrial carcinogenesis and are closely linked through metabolic and hormonal pathways (Zhong et al., 2025; Friberg et al., 2007; Nead et al., 2015; Wise et al., 2016; Renehan et al., 2010). Advancing age is also a well-established risk factor for endometrial carcinoma and has been included in previous preoperative prediction models (Vetter et al., 2020; Matsuo et al., 2015). Age was evaluated both as a continuous variable and dichotomized at 65 years. Given the intended clinical use of the nomogram, the dichotomous specification was selected for greater clinical interpretability and ease of use, with similar discrimination between the two specifications.

Diagnostic modality showed one of the strongest associations with concurrent carcinoma, with hysteroscopy-guided sampling or dilation and curettage associated with substantially lower odds of concurrent endometrial carcinoma compared with office endometrial biopsy. Hysteroscopy may allow more representative sampling through direct visualization and targeted biopsy of suspicious lesions (*Obstet. Gynecol.*, 2023; Di Spiezio Sardo et al., 2022; Sakna et al., 2023). However, this association should be interpreted cautiously, as the choice of sampling modality was clinically determined and may reflect patient selection, diagnostic workup patterns, or other unmeasured factors rather than the sampling method itself. Notably, the association remained consistent in the sensitivity analysis that included women with preoperative pathological suspicion of carcinoma.

Concurrent endometrial carcinoma was identified less frequently in our cohort than in most previous studies (Trimble et al., 2006; Doherty et al., 2020; Vetter et al., 2020). This difference may be explained, at least in part, by our exclusion of patients whose preoperative pathology already raised suspicion for malignancy. These patients represent a clinically distinct population in whom the pathological findings themselves are likely to influence preoperative management. By focusing on women with an unequivocal diagnosis of EIN without pathological suspicion of carcinoma, our cohort represents the population in whom additional preoperative risk stratification may be particularly relevant. When these women were included in a sensitivity analysis, the associations with type 2 diabetes mellitus and diagnostic modality remained generally consistent.

From a clinical perspective, improved preoperative estimation of concurrent endometrial carcinoma may provide additional information for patient counseling, gynecologic oncology referral, and surgical planning. Clinical characteristics, sampling information, and detailed pathological assessment may represent complementary components of preoperative risk stratification. The role of sentinel lymph node assessment in women with EIN remains an area of active investigation (Nahshon et al., 2024; Matanes et al., 2023; Catozzo et al., 2026). However, the proposed model should be considered an adjunct to clinical and pathological assessment rather than a tool for directing surgical management before prospective and external validation.

## 5 Strengths and limitations

This study has several strengths. We specifically focused on women with a preoperative diagnosis of EIN without pathological suspicion of carcinoma, representing a clinically relevant population in whom the residual risk of concurrent malignancy remains uncertain. Furthermore, the nomogram was developed using routinely available preoperative variables, and its performance was assessed using bootstrap internal validation together with ridge and Firth penalized regression sensitivity analyses.

Several limitations should also be acknowledged. This was a retrospective, single-center study with a relatively small sample size and a limited number of concurrent carcinoma events. The number of events relative to the number of predictors increases the risk of model overfitting despite the use of bootstrap validation and penalized regression sensitivity analyses. In addition, the diagnostic sampling modality was clinically determined and may reflect patient selection, diagnostic workup patterns, or other unmeasured factors, which may influence the observed association with concurrent carcinoma. Finally, the model was developed and internally validated within the same institution and requires prospective and external validation in larger, independent cohorts before its clinical utility can be established.

## 6 Conclusion

A simple preoperative nomogram based on routinely available clinical variables showed promising internally validated discrimination for estimating concurrent endometrial carcinoma in women with EIN without pathological suspicion of malignancy. Prospective and external validation in independent cohorts is required before clinical implementation.

## CRediT authorship contribution statement

**Gil Zeevi:** Writing – review & editing, Writing – original draft, Methodology, Formal analysis, Conceptualization. **Effi Yeoshoua:** Writing – review & editing, Methodology, Investigation, Conceptualization. **Chani Dahan:** Writing – review & editing, Investigation, Data curation. **Or Bercovich:** Writing – review & editing, Validation, Methodology, Formal analysis. **Oded Raban:** Writing – review & editing, Investigation, Data curation. **Ariella Jakobson-Setton:** Writing – review & editing, Visualization, Supervision, Investigation. **Gad Sabah:** Writing – review & editing, Supervision, Investigation. **Ran Matot:** Writing – review & editing, Investigation, Data curation. **Adi Borovich:** Writing – review & editing, Methodology, Data curation. **Ram Eitan:** Writing – review & editing, Writing – original draft, Supervision, Methodology, Investigation, Conceptualization.

## Ethics approval and consent to participate

The study was approved by the local institutional review board (approval no.0037–25). Informed consent was waived due to the retrospective study design.

## Funding

This research did not receive any specific grant from funding agencies in the public, commercial, or not-for-profit sectors.

## Declaration of competing interest

The authors declare that they have no known competing financial interests or personal relationships that could have appeared to influence the work reported in this paper.

## Appendix A Supplementary data

Supplementary material Study flow chart (Figure S1), sensitivity analysis of the multivariable logistic regression model using ridge and firth penalized regression (Table S1), and bootstrap-corrected calibration plot for predicting concurrent endometrial carcinoma (Figure S2).
