# Supplementary material for: Predicting concurrent endometrial carcinoma in women with endometrial intraepithelial neoplasia without preoperative suspicion of malignancy
Source: Gynecol Oncol Rep. 2026 Oct 19;67:102236. doi: 10.1016/j.gore.2026.102236 (PMC13716957; doi:10.1016/j.gore.2026.102236)
Supplement: Supplementary file 1 [file mmc1.docx]

**Supplementary Table S1. Sensitivity analyses of the multivariable logistic regression model using ridge and Firth penalized regression**

| **Predictor** | **aOR** | **p-value** | **Ridge - aOR** | | **Firth - aOR** |  |
| --- | --- | --- | --- | --- | --- | --- |
| Age ≥ 65 years | 3.97 | 0.054 | | 2.82 | 3.34 (0.98–13.74) | |
| Body mass index (kg/m2) | 1.07 | 0.112 | | 1.06 | 1.07 (0.98–1.16) | |
| Type 2 diabetes mellitus | 4.18 | 0.034 | | 3.06 | 3.88 (1.09–13.85) | |
| Diagnostic modality* | 0.11 | 0.004 | | 0.20 | 0.13 (0.03–0.53) | |

*Diagnostic modality: Hysteroscopy or Dilation and curettage vs Pipelle

aOR, Adjusted OR

**Supplementary Figure S2. Calibration plot of the final multivariable model for predicting concurrent endometrial carcinoma, with bootstrap correction**
